# Identifying lncRNAs and mRNAs related to survival of NSCLC based on bioinformatic analysis and machine learning

**DOI:** 10.18632/aging.205783

**Published:** 2024-05-01

**Authors:** Wei Yue, Jing Wang, Bo Lin, Yongping Fu

**Affiliations:** 1 Innovation Centre for Information, Binjiang Institute of Zhejiang University, Hangzhou 310053, China; 2 College of Computer Science and Technology, Zhejiang University, Hangzhou 310027, China; 3 Department of Cardiovascular Medicine, Affiliated Hospital of Shaoxing University, Shaoxing 312099, China

**Keywords:** NSCLC, survival, machine learning, CDC6, CEP55

## Abstract

Non-small cell lung cancer (NSCLC) is the most common histopathological type, and it is purposeful for screening potential prognostic biomarkers for NSCLC. This study aims to identify the lncRNAs and mRNAs related to survival of non-small cell lung cancer (NSCLC). The expression profile data of lung adenocarcinoma and lung squamous cell carcinoma were downloaded in The Cancer Genome Atlas (TCGA) and Gene Expression Omnibus (GEO) dataset. A total of eight survival related long non-coding RNAs (lncRNAs) and 262 survival related mRNAs were filtered. By gene set enrichment analysis, 17 significantly correlated Gene Ontology signal pathways and 14 Kyoto Encyclopedia of Genes and Genomes signal pathways were screened. Based on the clinical survival and prognosis information of the samples, we screened eight lncRNAs and 193 mRNAs by single factor Cox regression analysis. Further single and multifactor Cox regression analysis were performed, 30 independent prognostication-related mRNAs were obtained. The PPI network was further constructed. We then performed the machine learning algorithms (Least absolute shrinkage and selection operator, Recursive feature elimination, and Random forest) to screen the optimized DEGs combination, and a total of 17 overlapping mRNAs were obtained. Based on the 17 characteristic mRNAs obtained, we firstly built a Nomogram prediction model, and the ROC values of training set and testing set were 0.835 and 0.767, respectively. By overlapping the 17 characteristic mRNAs and PPI network hub genes, three genes were obtained: CDC6, CEP55, TYMS, which were considered as key factors associated with survival of NSCLC. The *in vitro* experiments were performed to examine the effect of CDC6, CEP55, and TYMS on NSCLC cells. Finally, the lncRNAs-mRNAs networks were constructed.

## INTRODUCTION

Lung cancer is one of the malignant tumors with the highest case fatality rate in the world. The incidence rate and mortality of lung cancer have been ascended globally, especially in a number of developing countries [1]. Non-small-cell lung cancer (NSCLC) is the most common histopathological type, accounting for about 85% of lung cancer. Lung adenocarcinoma (LUAD) and squamous cell carcinoma (LUSC) are two primary subtypes of NSCLC [2]. NSCLC begins with concealment and progresses rapidly. Most patients are diagnosed in local late stage or advanced stage, often losing the opportunity of the first radical resection. Due to the progress of comprehensive treatment methods for NSCLC, in addition to traditional radiotherapy, chemotherapy and other treatment methods, targeted therapy, immunotherapy and a variety of combined treatment modes have flourished. These strategies demonstrate extensive and significant clinical efficacy. However, the survival rate of NSCLC is still at a relatively low level. It has been reported that from 2012 to 2015, the survival rate of NSCLC in Chinese male patients was only 16.8%, 62.5% lower than that of thyroid cancer with the highest survival rate [3]. The survival rate of Chinese women with NSCLC is 25.1%, which is also classified as low survival rate [4]. Therefore, it is urgent to explore new strategies that can improve the clinical therapeutic effect of NSCLC.

Machine learning is a multi-disciplinary and interdisciplinary discipline, covering knowledge of probability theory, statistics, approximate theory and complex algorithms [5]. It uses computers as tools and is committed to simulating human learning methods, dividing existing content into knowledge structures to effectively improve learning efficiency, and integrating computer science and statistics into medical problems [6]. By improving algorithms, absorbing input data, applying computer analysis to predict the output value within the acceptable accuracy range, identifying the patterns and trends in the data, and finally learning from previous experience, the development of machine learning brings a new direction to the diagnosis and treatment of lung cancer [7]. Nomogram is the RMS package in the R statistical software, based on the LNR settings [8, 9]. The consistency index (C-index) and the calibration map were used to measure the performance of the model [10]. The consistency index is generally between 0.5-1. When the C index value is closer to 1, it is larger, but also indicates that the consistency of the model is better, that is, the prediction performance of the model is better.

In this study, we synthesized the expression profile data of LUAD and LUSC samples, screened the lncRNAs and mRNAs that are significantly related to survival, further screened the characteristic genes through different machine learning algorithms, and constructed the survival status classification model of the samples.

## MATERIALS AND METHODS

### Data processing

The LUAD and LUSC gene expression level data were downloaded from Xena Database (https://xenabrowser.net/datapages/), including 585 and 550 samples, respectively. The detection platform was Illumina HiSeq 2000 RNA Sequencing. According to the sample clinical information downloaded at the same time, the LUAD and LUSC samples with survival and prognosis information were retained. A total of 994 NSCLC tumor samples and 107 normal control samples were included in this analysis. Data from TCGA samples were used for training data sets. Since LUAD and LUSC are gene expression level data from different batches, we first use the R3.6.1 sva package [11] version 3.38.0 (http://www.bioconductor.org/packages/release/bioc/html/sva.html) to remove the batch effect of LUAD and LUSC expression profile data.

At the same time, data in GSE37745 [12, 13] of NSCLC expression profile were downloaded from the NCBI GEO database (https://www.ncbi.nlm.nih.gov/), including 196 NSCLC tumor samples with clinical prognosis information. The detection platform is GPL570 Affinemetrix Human Genome U133 Plus 2.0 Array. This data set was used as a validation set.

After downloading and obtaining the expression profile data, we annotated the detected lncRNAs and mRNAs according to the Transcript ID in the Illumina HiSeq 2000 RNA sequencing annotation platform for the TCGA platform detection data. For the NCBI GEO dataset, we download the detailed annotation information (including probe, gene symbol, RNA type and other information) of the platform involved in the corresponding platform from the Ensemble gene browser 96 database Biomart [14] (http://asia.ensembl.org/index.html), and then re-annotated the detection probe to obtain the corresponding expression level of the detected lncRNA and mRNA.

### Screening of differentially expressed RNAs (DERs)

In the expression profile data set after combining LUAD and LUSC, the samples were first divided into Tumor vs. Control comparison groups according to the sample source, and then in the tumor samples, the samples were divided into Dead vs. Alive comparison groups according to the survival status of the samples [15–17]. Later, we used the limma package Version 3.34.7 (https://bioconductor.org/packages/release/bioc/html/limma.html) to screen DERs in the two comparison groups by R3.6.1 language, and FDR<0.05 and | log2FC |>0.5 were selected as the threshold for screening significant factors.

Finally, we compared the DERs filtered in the comparison groups of Tumor vs. Control and Dead vs. Alive, and overlapping part was obtained. Based on DAVID version 6.8 [18, 19] (https://david.ncifcrf.gov/), GO Biological Process and KEGG signal pathway enrichment analysis was performed, and FDR<0.05 was selected as the threshold of enrichment significance.

### Identifying DERs with a significant association of prognosis

For the selected DERs, combined with the clinical survival and prognosis information of the samples, the single-factor Cox regression analysis of the survival package Version 2.41-1 in R3.6.1 (http://bioconductor.org/packages/survivalr/) was used to screen the significantly different expressions of lncRNAs and mRNAs that were significantly related to the survival and prognosis of NSCLC [20]. The NSCLC prognosis-related mRNAs were further analyzed by multivariate Cox regression, and the independent prognostic related mRNAs were selected. P-value less than 0.05 was selected as the threshold for screening significant correlation.

### Construction of protein-protein interaction (PPI) network and analysis of network topology

The STRING database [21] (Version:11.0, http://string-db.org/) was used to search for the interaction relationship between mRNAs gene product proteins that were significantly related to survival and prognosis, and the interaction network was constructed. The network was visualized and the network topology structure was analyzed through Cytoscape Version 3.6.1 [22] (http://www.cytoscape.org/).

### Optimal mRNAs screening and nomogram diagnostic model construction

Based on the expression level of mRNAs that were significantly related to the independent prognosis obtained from the previous screening, three different optimization algorithms [LASSO (least absolute shrinkage and selection operator) [23], RFE (recursive feature elimination) [24], and RF (random forest) [25]] were used to screen the characteristic factors. We subsequently compared the results of the three algorithms and selected the overlapping part as the final feature mRNAs combination.

### Construction and verification of nomogram diagnostic model

We used R3.6.1 rms package (https://cran.r-project.org/web/packages/rms/index.html) Version 5.1-2 to build the Nomogram model [26], and analyzed the model with a line chart, using C index as a parameter to measure the fit between the model and the actual. Based on the selected characteristic mRNA factors, we used the R3.6.1 language rmda package [27] (https://cran.r-project.org/web/packages/rmda/index.html) Version 1.6 to observe the model yield. Finally, in the validation data from GSE37745, the Nomogram model was also constructed based on the characteristic mRNA factors obtained as previously screened to validate the diagnostic model efficacy. The ROC curves of the Nomogram model were assessed by R3.6.1 pROC v1.18.0 package [28] (https://cran.r-project.org/web/packages/pROC/index.html).

### Screening of the key mRNAs

We compared the selected characteristic genes for constructing survival diagnosis model with the important link hub genes in PPI network, and selected the overlapping part as the important factor. In the combined TCGA training set and GSE37745 validation data set, the Kaplan-Meier curve method in the survival package Version 2.41-1 in R3.6.1 [20] was used to analyze and display the correlation between the expression level of important genes and survival prognosis.

### Co-expression network of lncRNAs-mRNAs

Based on the characteristic factors in the diagnosis model of lncRNAs and Nomogram, which were significantly correlated with independent prognosis, the Pearson correlation coefficient between them was calculated by using the cor function (http://77.66.12.57/R-help/cor.test.html) in R3.6.1, and the co-expression network of lncRNAs-mRNAs with independent prognosis was constructed. The network was displayed through Cytoscape Version 3.6.1.

### Cell lines

The NSCLC cell lines (A549 and H1299) were purchased from the Cell Bank of Chinese Academy of Medical Science (Shanghai, China). A549 and H1299 cells were separately cultured in the Dulbecco’s modified eagle medium (DMEM) supplemented with 10% fetal bovine serum (FBS), 100 U/mL penicillin and 100 mg/mL streptomycin and incubated in an incubator at 37° C and 5% CO_2_ conditions.

### Cell transfection

A549 and H1299 cells were harvested to prepare cell suspension with the serum-free DMEM, with the final cell concentration of 1 × 10^6^ cells/mL. Thereafter, the cell suspension (1 ml/well) was added into the 6-well plates. Then, the siRNAs targeted *CDC6* (siCDC6-1, siCDC6-2), *CEP55* (siCEP55-1, siCEP55-2), *TYMS* (siTYMS-1, siTYMS-2), and negative control (NC, Genechem, Shanghai, China) were transfected into A549 and H1299 cells, respectively, following the manuals. Lipofectamine 2000 (Thermo Fisher Scientific, Waltham, MA, USA) was used in cell transfection, and all cells were transfected for 8 h under 37° C and 5% CO_2_ conditions. Afterwards, DMEM supplemented with 10% FBS was added into each well to culture cells.

### RT-PCR

The extraction of total RNA in A549 and H1299 cells was performed by using the Trizol Reagent (Life Technologies, Shanghai, China) according to the instructions. The total RNA was reverse transcribed into the cDNA template by using the PrimeScript™ RTreagent kit (Takara, Beijing, China). The expression of *CDC6*, *CEP55*, and *TYMS* mRNA was scrutinized by qPCR with the SYBR Premix Ex Taq™ kit (Takara, Dalian, China) on the ABI 7900HT Real-Time PCR system (Applied Biosystems, Foster City, CA, USA). The relative mRNA expression of *CDC6*, *CEP55*, and *TYMS* was estimated by the 2^-ΔΔCt^ method and normalized to GAPDH.

### Western blot

The total proteins in A549 and H1299 cells were extracted by applying the RIPA lysis buffer (Beyotime, Shanghai, China) in the light of the instructions. The concentration of total protein samples was scrutinized by using the BCA kit (Beyotime, Shanghai, China). A weight of 20 μg of the total protein sample were collected and separated with 10% SDS-PAGE. After the blockage by 5% skimmed milk for 1 h at 25° C, the proteins were transferred to the polyvinylidene difluoride (PVDF) membranes. Rabbit anti-primary antibodies were then dropped onto the PVDF membranes to probe the proteins for 12 h at 4° C, including anti-CDC6 (1:1000, ab109315, Abcam, Shanghai, China), anti-CEP55 (1:1000, ab170414, Abcam), anti-TYMS (1:1000, CSB-PA025393GA01HU, CUSABIO, Wuhan, China) and anti-GAPDH (1:1000, CSB-MA000071, CUSABIO). Followed by this, goat anti-rabbit secondary antibody (1:2000, A21020, AmyJet Scientific, Wuhan, China) was utilized for 2 h treatment of the proteins at room temperature. The enhanced chemiluminescent (ECL) kit (AmyJet Scientific, Wuhan, China) was applied for the visualization of the specific protein blots according to the directions. The quantification of proteins was determined by Image Lab software 3.0 (Bio-Rad Laboratories, Hercules, CA, USA).

### Cell counting kit-8 (CCK-8) assay

CCK-8 assay was performed to estimate cell viability of A549 and H1299 cells. In brief, 1 × 10^4^ A549 and H1299 cells were respectively seeded into the 96-well plates containing 100 μL DMEM supplemented with 10% FBS, and maintained under 37° C and 5% CO_2_ conditions. After 24, 48, 72 and 96 h of culture, the 96-well plates were taken out from the incubator. Then, CCK-8 solution (10 μL/well) was added into each well to incubate cells for 4 h at 37° C. The absorbance (OD) value of each well was measured at 450 nm using a microplate reader.

### Cell migration and invasion

The 24-well Transwell chambers (Litchi Biotechnology, Shanghai, China) were purchased for evaluating cell migration and invasion. A549 and H1299 cells were suspended into 300 μL non-serum DMEM, followed by being seeded into the upper chambers. DMEM containing 10% FBS was added into the lower chambers. After 24 h of incubation, the migration cells were sequentially fixed by 4% paraformaldehyde and stained by 1% crystal violet for 10 min. The number of migration cells was counted under the microscope (IX81, Olympus, Tokyo, Japan). For cell invasion, 100 μL Matrigel (Litchi Biotechnology) was pre-coated into the upper chambers before cell seeding.

### Cell apoptosis

A549 and H1299 cells were harvested and washed by PBS twice. A549 and H1299 cells were subsequently resuspended into 100 μL 1× Binding Buffer. Then 5 μL FITC and 10 μL propidium iodide solution were added and gently mixed. Cells were placed at 25° C for 15 min. A total of 400 μL 1× Binding Buffer was then added to treated cells for 15 min on ice. Cell apoptosis was analyzed by the FACSCalibur flow cytometer (BD Biosciences, San Jose, CA, USA).

### Statistical analysis

The experiments in the present study were carried out in triplicates. Statistical analysis was implemented using GraphPad Prism 10 software. The data were displayed as mean ± standard deviation. Paired Student’s t-test was executed to analyze the difference between the two groups. One-way analysis of variance and Tukey’s post hoc test were employed for the data comparison in more than two groups. *P* < 0.05 revealed a statistically significant difference.

### Availability of data and material

The datasets generated and/or analysed during the current study are available from the corresponding author on reasonable request.

## RESULTS

### Screening of factors with a significant association of prognosis

**Figure 1:**
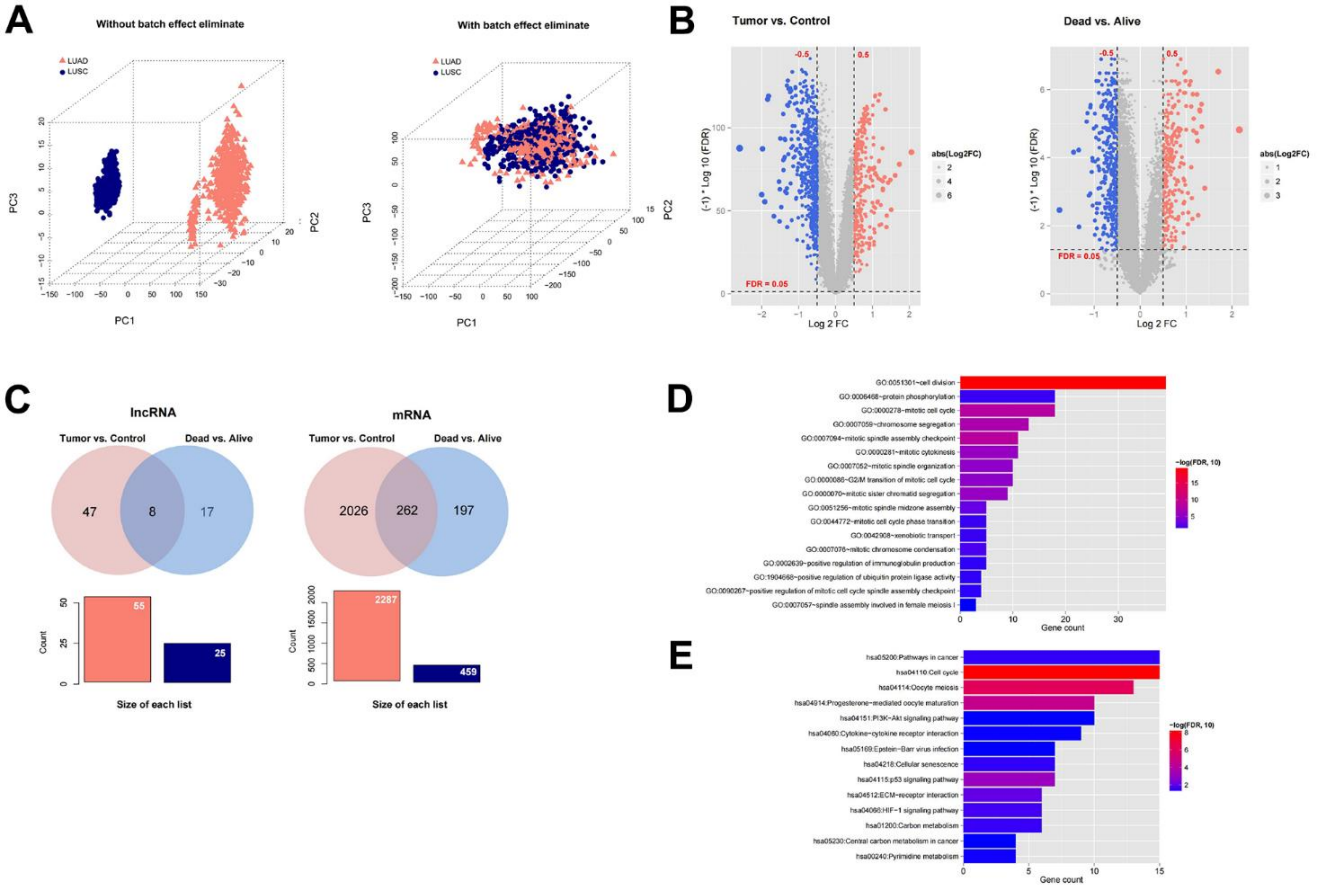
**Screening prognosis-related mRNAs and lncRNAs based on TCGA data.** (**A**) The sample relationship before and after batch effect removal. (**B**) Heatmap of differentially expressed mRNAs and lncRNAs in Tumor (994) vs Control (107) comparison group and Dead (394) vs Alive (600) comparison group. (**C**) A total of eight overlapping lncRNAs and 262 mRNAs were filtered. (**D**–**E**) The enrichment analysis of GO function and KEGG signal pathway based on DAVID was carried out for the overlapped mRNAs with significant differential expression.

We downloaded the expression profile data corresponding to LUAD and LUSC in TCGA, and then combined them into a data set. The sample relationship before and after batch effect removal was shown in Figure 1A. In the expression profile data set after combining LUAD and LUSC, we first divided the samples into Tumor (n = 994) vs Control (n = 107) comparison group, and then divided the samples into Dead (n = 394) vs Alive (n = 600) comparison group. In the comparison group of Tumor vs. Control, 55 significantly differentially expressed lncRNAs and 2287 significantly differentially expressed mRNAs were screened (Figure 1B). In the Dead vs. Alive comparison group, 22 significantly differentially expressed lncRNAs and 459 significantly differentially expressed mRNAs were screened (Figure 1B). We further compared the DERs filtered in Tumor vs. Control and Dead vs. Alive, and a total of eight overlapping lncRNAs and 262 mRNAs were filtered (Figure 1C). The information list was shown in Supplementary Table 1. Finally, the enrichment analysis of GO function and KEGG signal pathway based on DAVID was carried out for the overlapped mRNAs with significant differential expression. A total of 17 significantly correlated GO signal pathways and 14 KEGG signal pathways were screened, which were displayed in Figure 1D–1F.

### Identifying of DERs with a significant association of prognosis and PPI network construction

**Figure 2:**
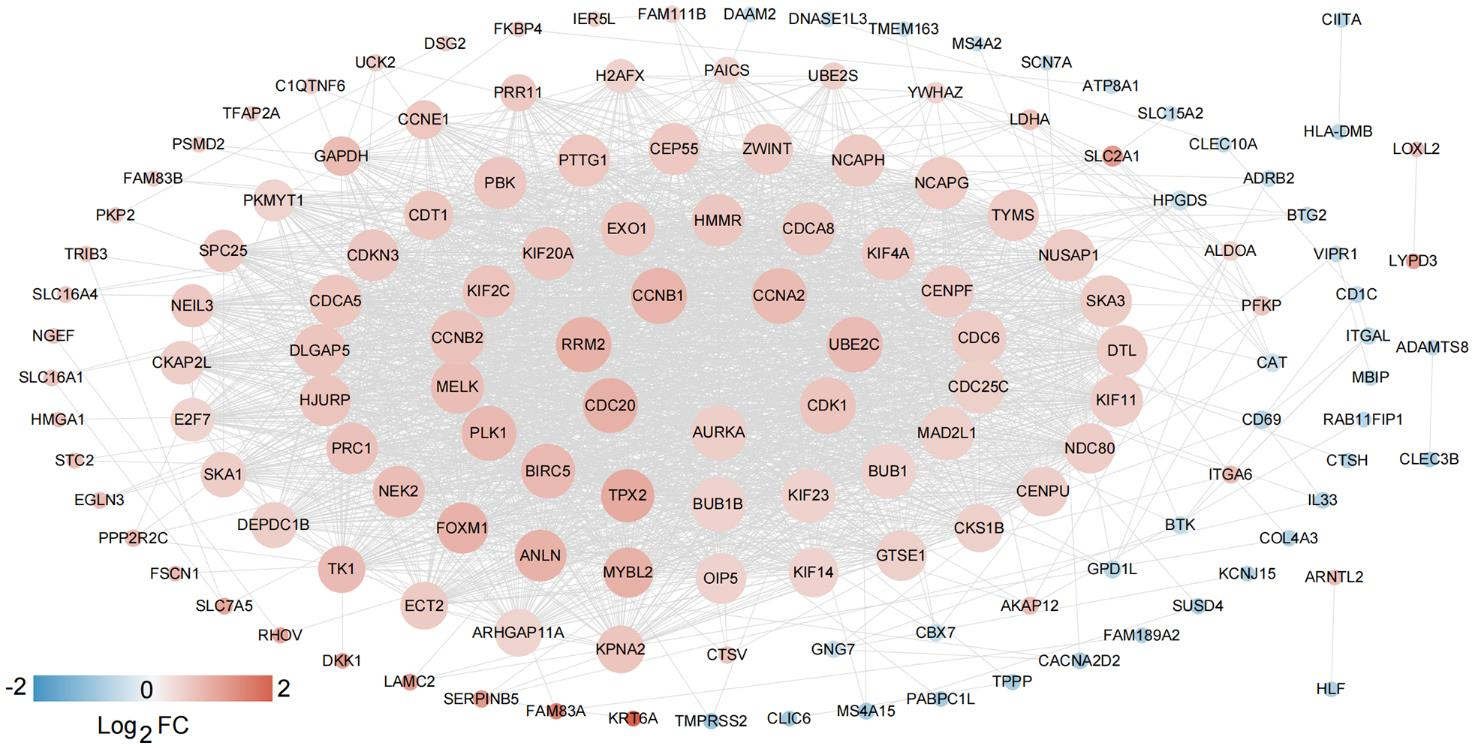
**PPI network construction.** The network contained 145 gene nodes in total.

Based on the clinical survival and prognosis information of the samples, eight lncRNAs and 262 mRNAs by single factor Cox regression analysis were screened. Eight lncRNAs and 193 mRNAs that were significantly related to survival and prognosis were obtained. The eight lncRNAs were: *FEZF1-AS1*, *SNHG12*, *BANCR*, *SNHG3*, *HLA-DQB1-AS1*, *SH3BP5-AS1*, *VIM-AS1*, and *FAM83A-AS1*. Further multifactor Cox regression analysis was performed on 193 prognostication-related mRNAs, and the independent prognostication-related mRNAs were selected. A total of 30 independent prognostication-related mRNAs were obtained, which was shown in Supplementary Table 2. The STRING database was used to search for the interaction relationship between 262 mRNAs product proteins that are significantly related to survival and prognosis. The PPI network was constructed, as shown in Figure 2. The network contained 145 gene nodes in total.

### Optimal mRNA marker excavation and nomogram diagnostic model construction

**Figure 3:**
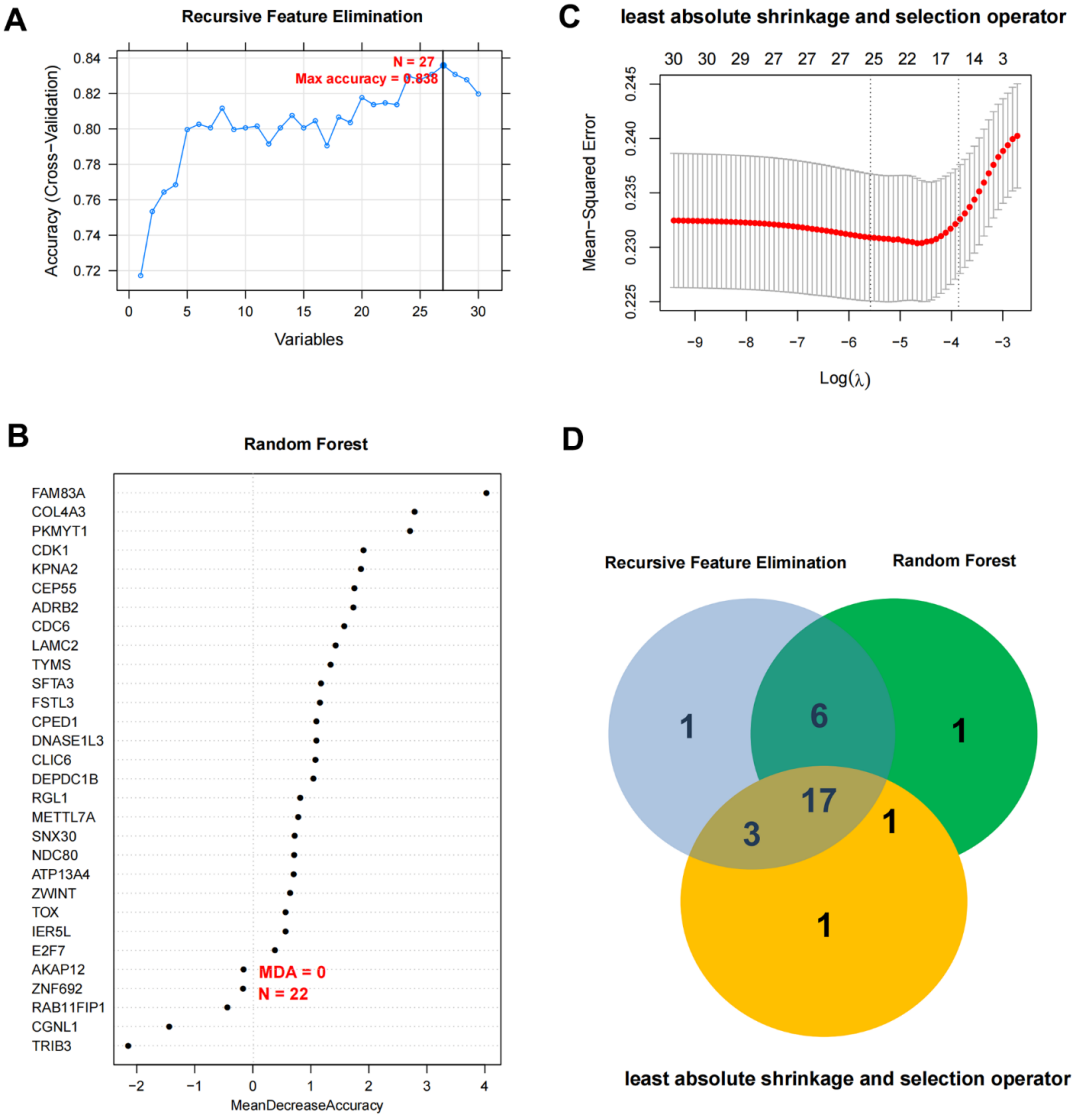
**Optimal mRNA marker excavation and nomogram diagnostic model construction.** (**A**–**C**) Filter characteristic mRNAs parameter diagram of RFE, RF, and LASSO. (**D**) Comparison chart of characteristic mRNAs combinations filtered by RFE, RF, and LASSO.

Based on the expression level of 30 independent prognosis significantly correlated mRNAs obtained in the previous step in the TCGA combined data set, the LASSO, RFE and RF algorithms were used to screen the optimized DEGs combination, and the parameter diagram of algorithm filtering is shown in Figure 3A–3C). In LASSO, RFE and RF algorithms, we screened 22, 27 and 25 mRNAs respectively. Comparing these three mRNAs sets, a total of 17 overlapping mRNAs were obtained (Figure 3D). The 17 mRNAs obtained as the final optimized mRNAs combination: *ADRB2*, *ATP13A4*, *CDC6*, *CEP55*, *CLIC6*, *COL4A3*, *CPED1*, *DEPDC1B*, *DNASE1L3*, *E2F7*, *FAM83A*, *FSTL3*, *IER5L*, *LAMC2*, *SFTA3*, *TOX*, *TYMS*.

### The diagnostic nomogram model construction and validation

**Figure 4:**
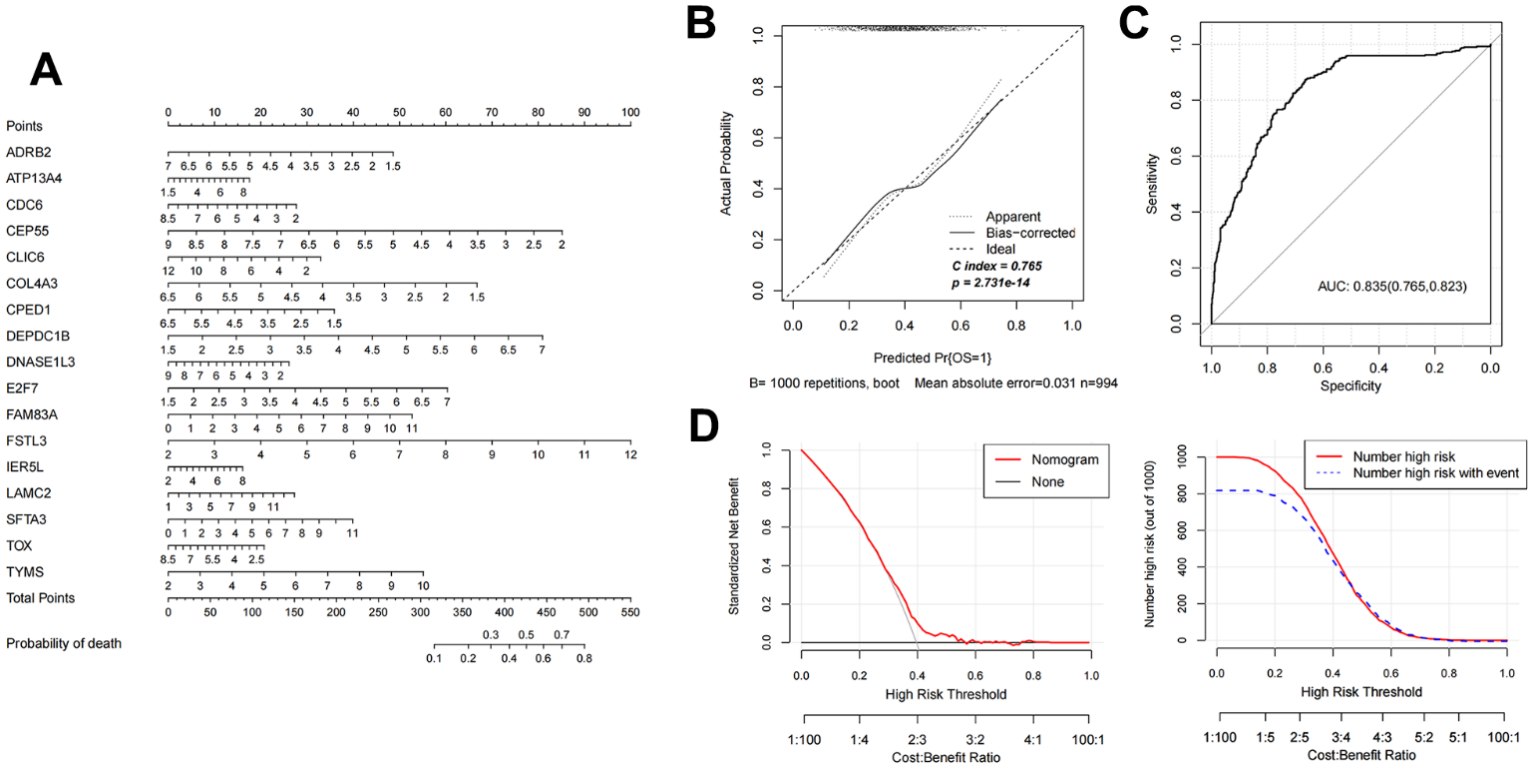
**Nomogram diagnostic model construction and evaluation.** (**A**) Nomogram model diagram based on the expression level of 17 characteristic mRNAs in the combined training data set. (**B**) Nomogram diagnostic model line chart. (**C**) The ROC value was calculated. (**D**) Model decision line diagram.

**Figure 5:**
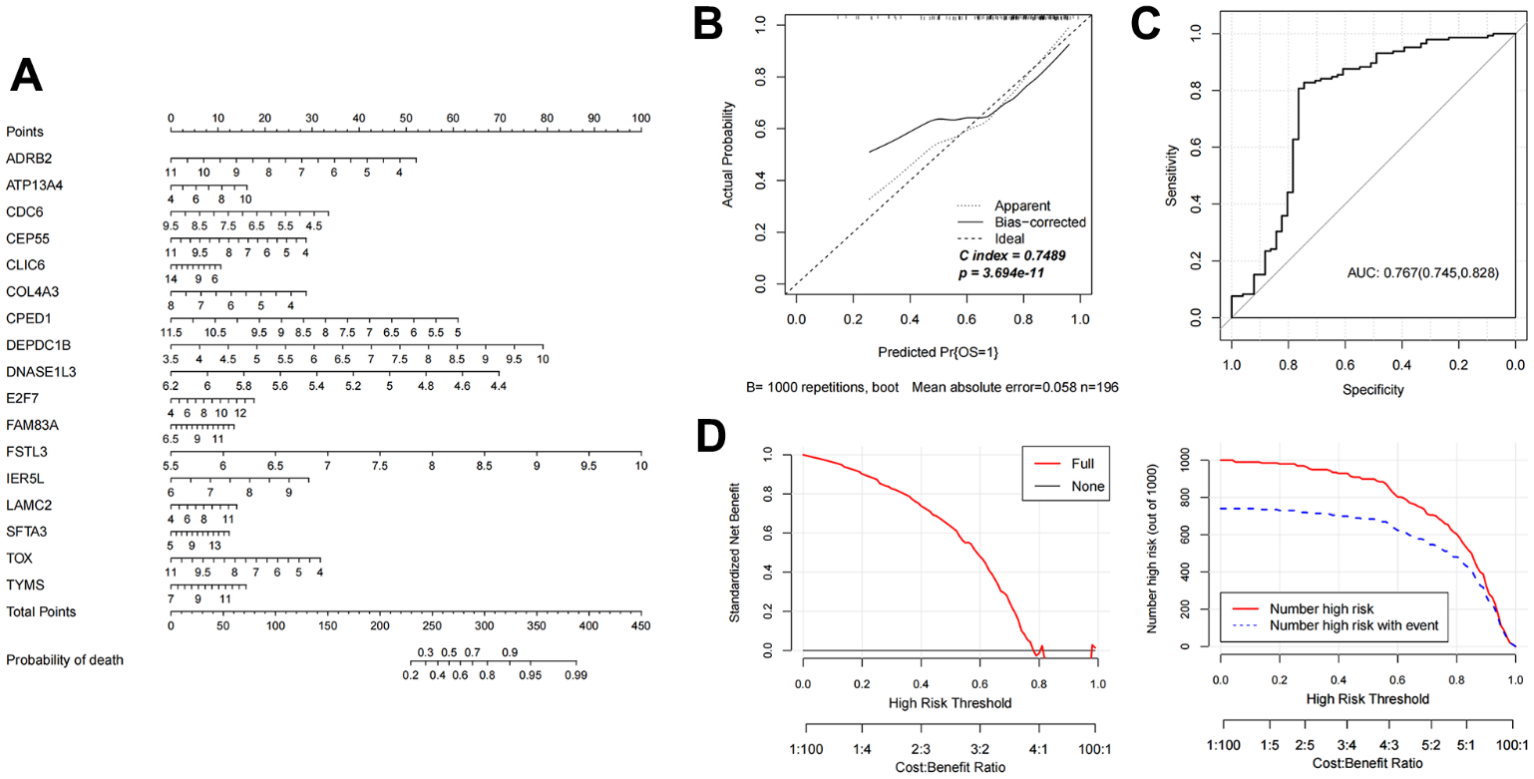
**Evaluation of nomogram diagnostic model in GSE37745 dataset.** (**A**) Nomogram model diagram of expression level of mRNAs in GSE37745 validation data set based on 17 features. (**B**) Nomogram diagnostic model line chart. (**C**) The ROC value was calculated. (**D**) Model decision line diagram.

Based on the 17 characteristic mRNAs obtained by screening, we constructed a Nomogram prediction model according to the expression level of each factor, as shown in Figure 4A. Then, the Nomogram diagnostic model was analyzed by line graph, as shown in Figure 4B, from which the Cindex value was 0.765. After that, the decision curve analysis was carried out on the model to observe the net return rate of the sample diagnosis results of the model, as shown in Figure 4D. In addition, the ROC curve of the model was analyzed, and the results are shown in Figure 4C. Finally, in the validation data set GSE37745, the Nomogram model was also built based on the 17 mRNAs factors screened previously to verify the effectiveness of the diagnostic model. The results were shown in Figure 5A–5D). The ROC values of training set and testing set were 0.835 and 0.767, respectively.

**Figure 6:**
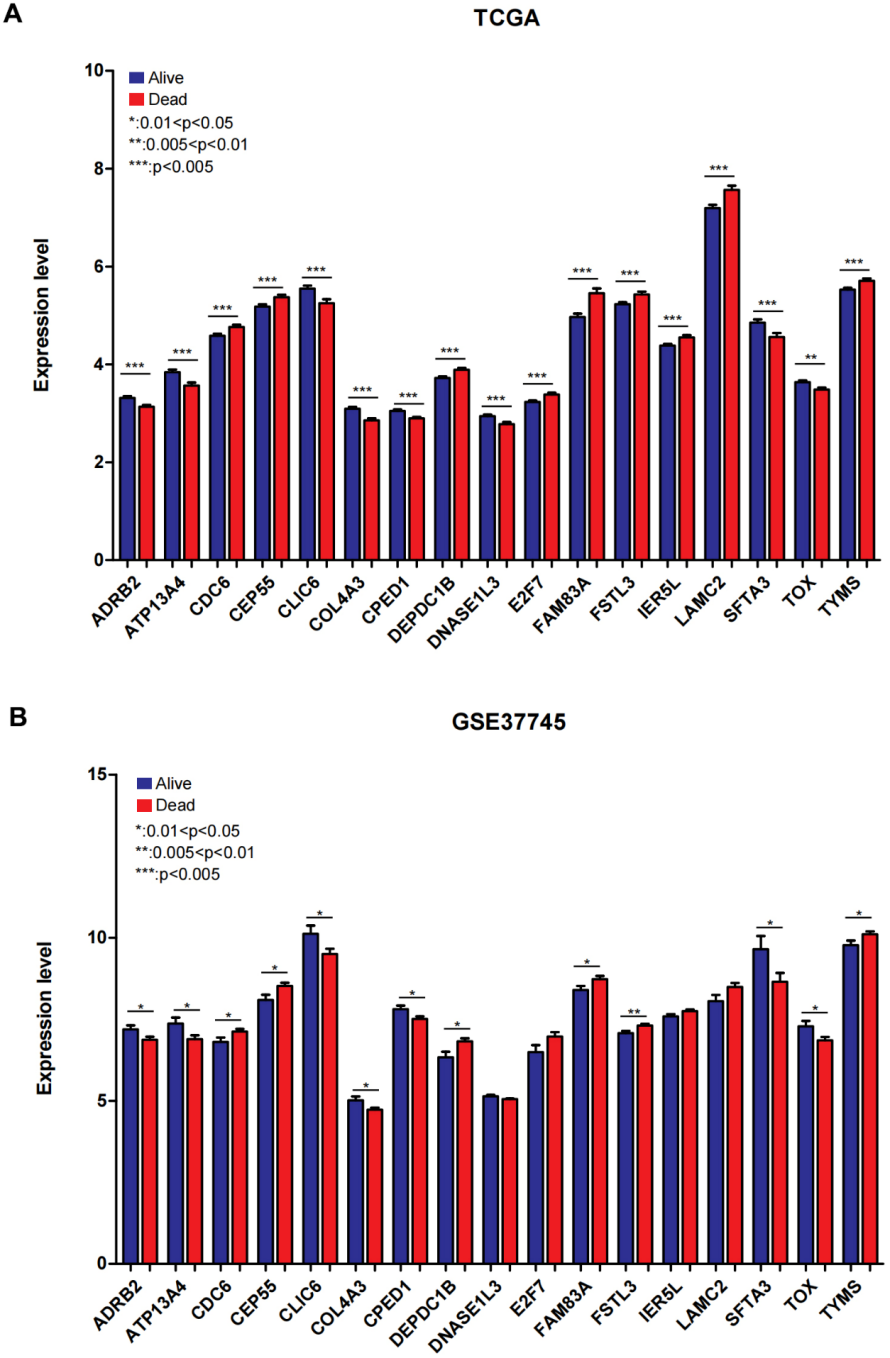
**The expression of 17 mRNAs in combined TCGA training set and GSE37745 testing dataset.** (**A**) The expression of 17 mRNAs in combined TCGA training set. (**B**) The expression of 17 mRNAs in GSE37745 dataset. 0.01<*P<0.05; 0.005< **P<0.01; ***P<0.005.

We visualized the expression level of 17 genes in the combined TCGA training data set and validation data set (GSE37745). As displayed in Figure 6A, 6B), the expression level of 17 genes in the GSE37745 validation data set was completely consistent with the direction of the expression difference in the combined TCGA training data set. The expression level of 13 genes, including *ADRB2*, *ATP13A4*, *CDC6*, *CEP55*, *CLIC6*, *COL4A3*, *CPED1*, *DEPDC1B*, *FAM83A*, *FSTL3*, *SFTA3*, *TOX*, *TYMS*, was significantly different in the group comparison.

### Screening of key genes

**Figure 7:**
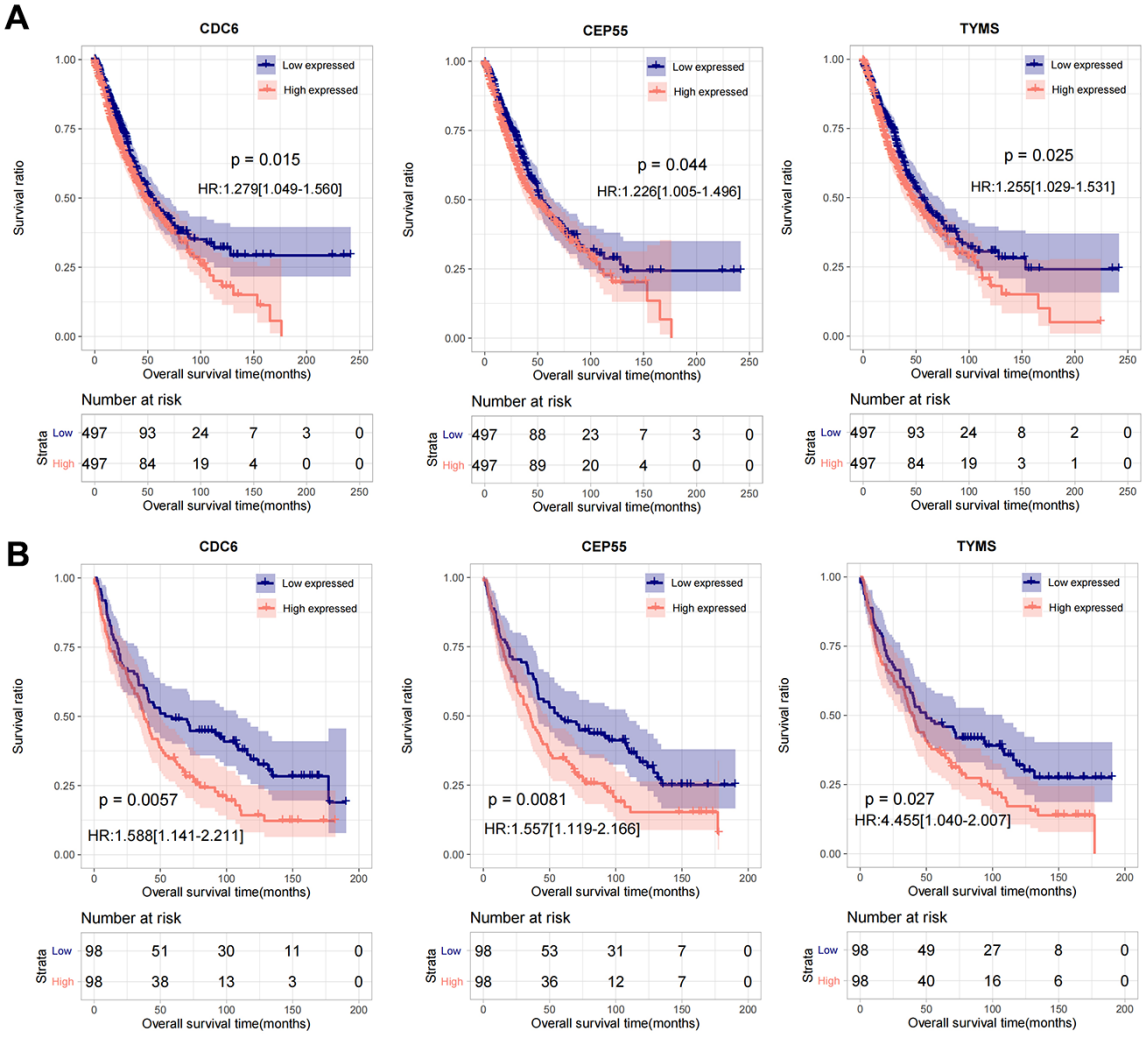
**The prognostic analysis of CDC6, CEP55, and TYMS in TCGA and GSE37745.** (**A**) Kaplan-Meier used for prognostic analysis of CDC6, CEP55, and TYMS in combined TCGA training set. (**B**) Kaplan-Meier used for prognostic analysis of CDC6, CEP55, and TYMS in GSE37745 validation data set.

By comparing the 17 characteristic genes selected to construct the survival diagnosis model with the important link hub genes in the PPI network, the overlapping part was selected as an important factor, and a total of 3 genes were obtained: *CDC6*, *CEP55*, *TYMS*. In the combined TCGA training set and GSE37745 validation data set, the samples were divided into low-volume (expression level lower than the median value) and high-volume expression group (expression level higher than or equal to the median value) according to the respective expression level of the three genes. The Kaplan-Meier curve method was used to analyze and display the correlation between the expression level of important genes and survival and prognosis. The results are shown in Figure 7A, 7B), high expression of *CDC6*, *CEP55*, and *TYMS* predicted poor prognosis.

### Construction of a co-expression network based on characteristic mRNAs and lncRNAs

**Figure 8:**
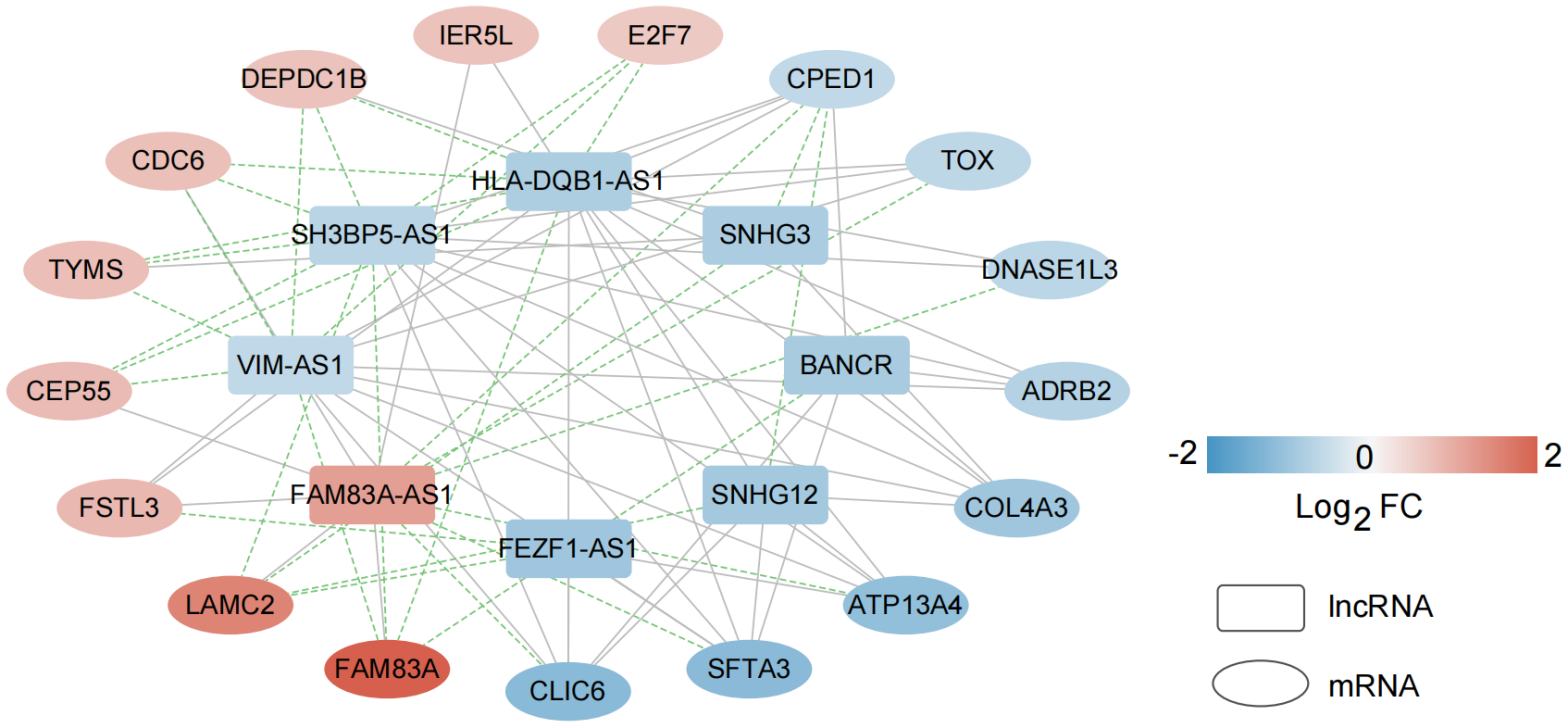
**Construction of a co-expression network based on characteristic mRNAs and lncRNAs.** A total of 79 pairs of relationship pairs were screened, and the relationship connection network was constructed.

Based on the expression level of eight lncRNAs that are significantly related to independent prognosis and 17 important characteristic genes related to survival status diagnosis, the expression correlation between them was calculated. After retaining the action pairs with significant correlation *P* < 0.05, a total of 79 pairs of relationship pairs were screened, and the relationship connection network is shown in Figure 8.

### CDC6, CEP55, and TYMS affected cell activities in NSCLC cells

NSCLC cells were transfected with siCDC6, siCEP55, and siTYMS, and the efficiency of transfection was examined by RT-PCR and western blot (Supplementary Figure 1). The siCDC6-1 (siCDC6), siCEP55-1 (siCEP55), and siTYMS-1 (siTYMS) showed favorable transfection efficiency, which were used for the further experiments. CCK8, Transwell and FCM were employed to test cell proliferation, migration, invasion and cell apoptosis. The result showed that siCDC6, siCEP55, and siTYMS inhibited cell proliferation, migration, and invasion in NSCLC cells, and promoted cell apoptosis in NSCLC cells (Supplementary Figures 2–4).

## DISCUSSION

The prognosis of lung cancer is poor, and the 5-year survival rate after diagnosis is only 16.2% [29]. Because of the lack of clinical symptoms in the early stage of the disease, when symptoms appear, the preferably treatment opportunity has been missed. In China, on the account of economic conditions and national awareness, there are very few lung cancer patients who can be diagnosed in the early stage [30, 31]. Therefore, early screening of NSCLC has great scientific significance. How to improve the accurate diagnosis, treatment and survival prognosis of NSCLC is particularly important. The TCGA project was started in 2006 by National Cancer Institute and National Human Genome Research Institute. It is an epoch-making project in the field of cancer genomics, which contains research data from different disciplines and institutions. TCGA has identified more than 20000 primary cancers at the molecular level and matched 33 normal tissue samples of cancer [32, 33]. Through the comprehensive analysis of genome, transcriptome and proteome data, valuable biological information about tumor molecular changes can be obtained.

We downloaded the RNA sequence data of LUAD and LUSC from TCGA database. By preliminary analysis, a total of eight survival related long non-coding RNAs (lncRNAs) and 262 survival related mRNAs were filtered. By gene set enrichment analysis, 17 significantly correlated GO pathways and 14 KEGG signal pathways were screened. The GO pathways like GO:0051301-cell division [34], GO:0006468-protein phosphorylation [35], and GO:0000278-mitotic cell cycle [36], which have been reported to exhibit an important role in the occurrence, development, drug resistance and metastasis of NSCLC. The KEGG pathways including hsa05200-Pathways in cancer [37], hsa04110-Cell cycle [38], and hsa04151-PI3K-Akt [39] signaling pathways are also reported to regulate the occurrence and development of NSCLC.

In view of the clinical survival and prognosis information of the samples, we screened eight lncRNAs and 193 mRNAs by single factor Cox regression analysis. Further single and multifactor Cox regression analysis were performed, 30 independent prognostication-related mRNAs were obtained. The PPI network was further constructed. The top ten hub genes were *CDK1*, *CCNB1*, *UBE2C*, *RRM2*, *CCNA2*, *AURKA*, *PLK1*, *CDC6*, and *CDC20*, most mRNAs of which have been reported to be involved in the regulation of cell cycle in lung cancer cells [40–43]. The machine learning algorithms (LASSO, RFE, and RF) were employed to screen the optimized DEGs combination, and a total of 17 overlapping mRNAs were obtained. Based on the 17 characteristic mRNAs obtained, we firstly built a Nomogram prediction model. The ROC values of training set and testing set were 0.835 and 0.767 respectively, which suggested that Nomogram prediction model represented favourable performance. By overlapping the 17 characteristic mRNAs and PPI network hub genes, three genes were obtained: *CDC6*, *CEP55*, *TYMS*, which was considered as key factors associated with survival of NSCLC. Allera-Moreau et al. reported that CDC6 was associated with overall, disease-free and relapse-free survival in NSCLC [44]. Another study indicated that *CDC6* was involved in the replication licensing and the proliferation, migration, and invasion of lung cancer cells mediated by miR-26a and miR-26b, and *CDC6* represented potential cancer diagnostic and prognostic markers as well as anticancer targets [45]. Centrosome-associated protein 55 kDa (CEP55) is a member of the coiled-coil protein family. Its main function is to anchor microtubule polymerization-associated protein, participate in spindle formation, and then regulate cell proliferation [46]. The protein is expressed in normal tissues and tumor cells, and CEP55 can be coupled with the centrosome and intermediates in the cell cycle. After phosphorylation, it plays a role in regulating the cell cycle. The overexpression of *CEP55* is significantly correlated with the tumor stage, invasion and metastasis of many malignant tumors [47]. Jiang et al. found that *CEP55* expression was commonly elevated in NSCLC tissues and overexpression of *CEP55* was correlated with unfavorable prognosis in the patients with NSCLC [48]. Fan et al. reported that *CEP55* expression affected the survival and prognosis of patients with NSCLC, and participated in the process of tumor immune response [49]. Moreover, thymidylate synthetase (TYMS) silencing was reported to increase the pemetrexed sensitivity of NSCLC cells [50]. Zhang et al. demonstrated that significant correlation was observed in *TYMS* expression and clinical features, especially histology in NSCLC [51]. Tsyganov et al. demonstrated that exploring *TYMS* expression could contribute to the personalized chemotherapy, which can improve treatment efficacy and reduce unnecessary toxicity [52]. In the present study, A549 and H1299 cells were transfected with siCDC6, siCEP55, and siTYMS, respectively. Cell proliferation, migration, invasion and apoptosis were examined. The results presented that silencing of *CDC6*, *CEP55*, *TYMS* showed carcinostatic effect on NSCLC cells. Finally, the lncRNAs-mRNAs networks were constructed, and a total of 79 pairs of relationship pairs were screened. There are some limitations in the present study. The relationship between lncRNAs and mRNAs and immune cells, the analysis of drug sensitivity data, and the analysis of microRNAs data based on public databases are not studied in the present study. These works can be part of our future work.

In conclusion, this study explored the lncRNAs and mRNAs related to survival of NSCLC based on bioinformatic analysis and machine learning. We firstly built a Nomogram prediction model, which exhibited favourable performance. *CDC6*, *CEP55*, and *TYMS* are considered as key factors associated with survival of NSCLC. This paper provides a new idea for the early screening of NSCLC.

## Supplementary Material

Supplementary Figures

Supplementary Table 1

Supplementary Table 2
