# Supplementary material for: Identifying lncRNAs and mRNAs related to survival of NSCLC based on bioinformatic analysis and machine learning
Source: Aging (Albany NY). 2024 May 1;16(9):7799–817. doi: 10.18632/aging.205783 (PMC11131976; doi:10.18632/aging.205783)
Supplement: Supplementary Figures [file aging-16-205783-s001.pdf]

SUPPLEMENTARY FIGURES

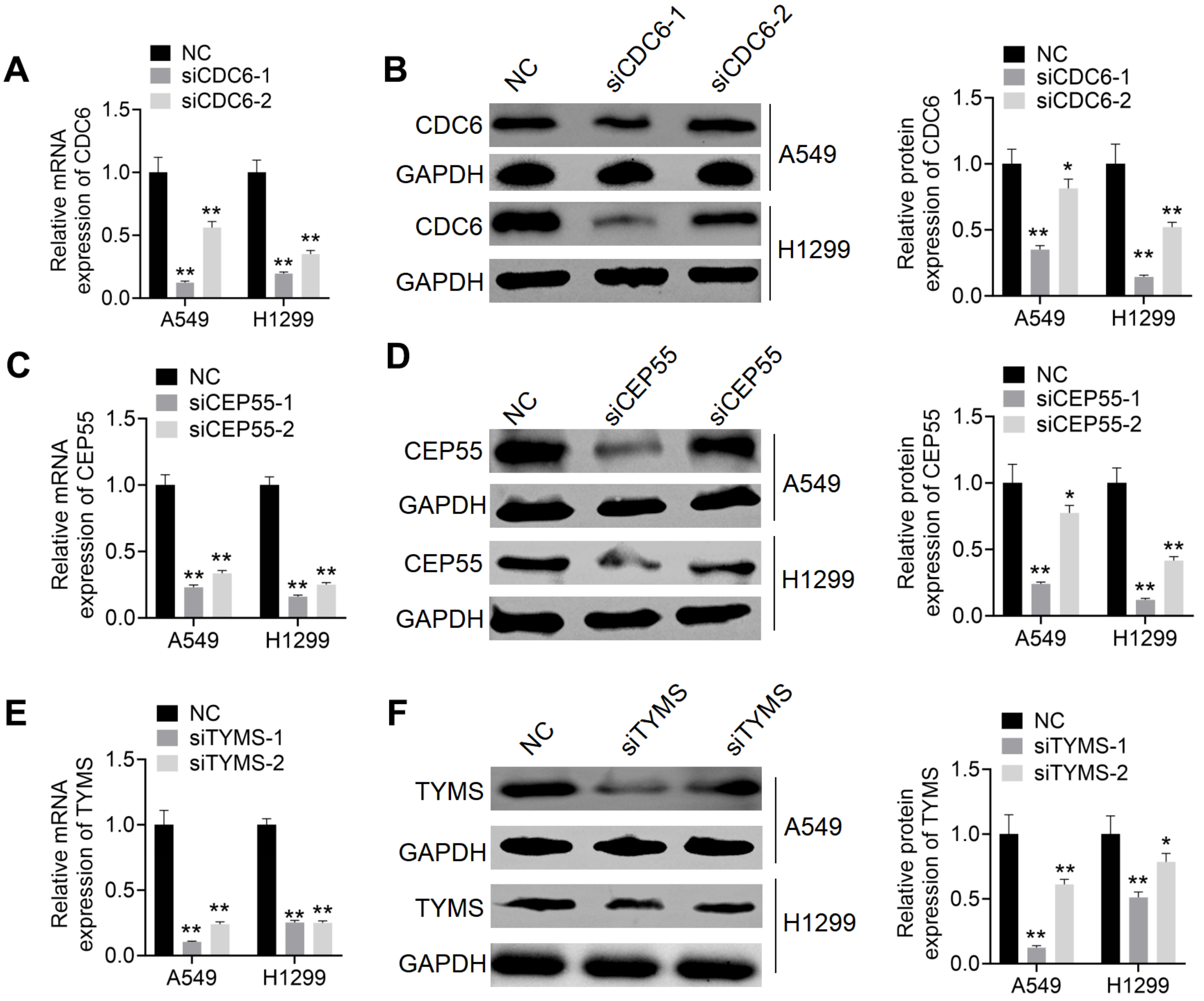

Supplementary Figure 1. Transfection efficiency was examined by PCR and western blot. (**A**, **B**) RT-PCR and western blot were performed to examine the transfection efficiency of CDC6 knockdown. (**C**, **D**) RT-PCR and western blot were performed to examine the transfection efficiency of CEP55 knockdown. (**E**, **F**) RT-PCR and western blot were performed to examine the transfection efficiency of TYMS knockdown. *P<0.05, **P<0.01 vs NC group.

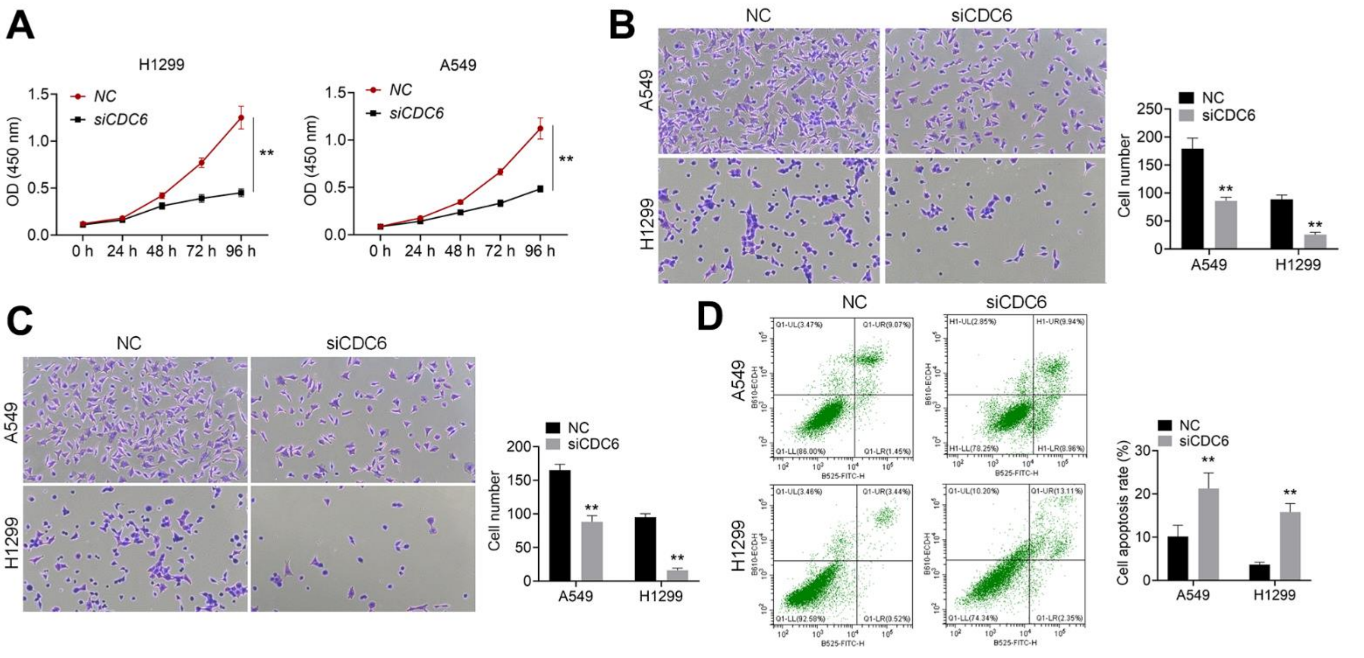

Supplementary Figure 2. Effect of CDC6 knockdown on malignant oncology phenotype in NSCLC cells. (**A**) Cell proliferation was examined by CCK8. (**B**, **C**) Cell migration and invasion were tested by Transwell assay. (**D**) Cell apoptosis was evaluated by FITC. **P*<0.05, ***P*<0.01 vs NC group.

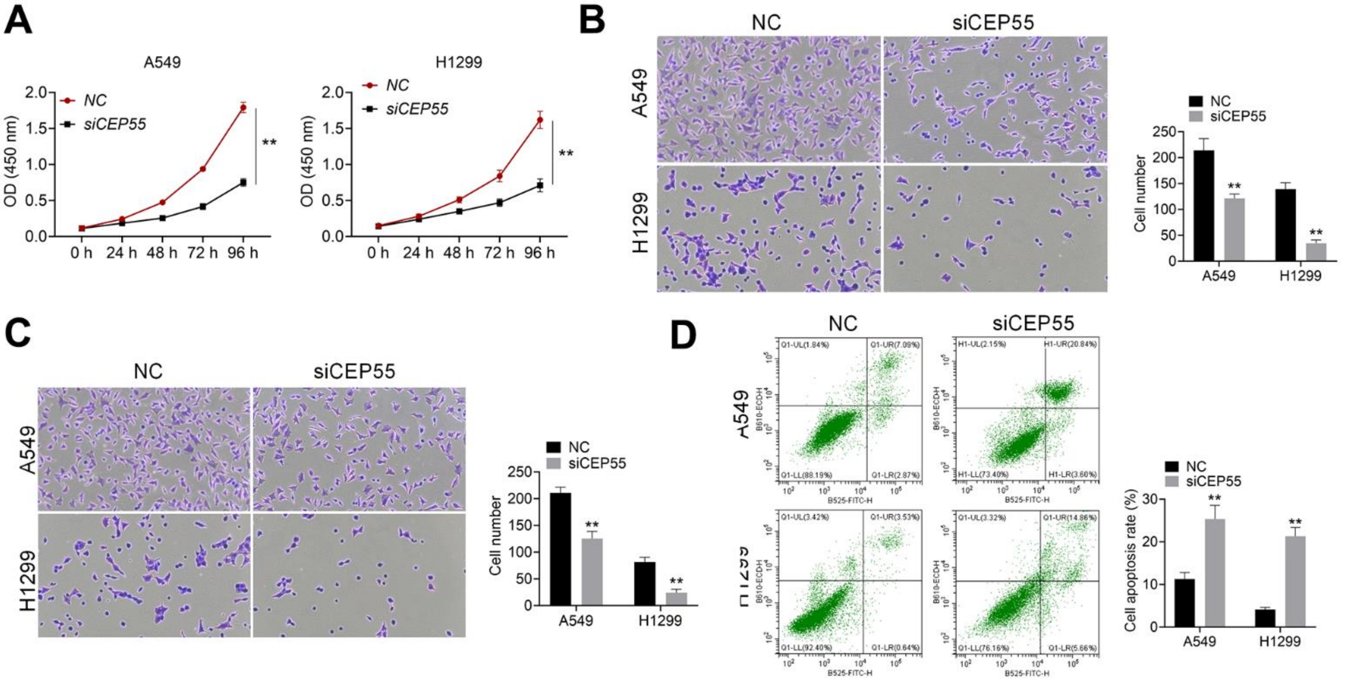

Supplementary Figure 3. Effect of CEP55 knockdown on malignant oncology phenotype in NSCLC cells. (**A**) Cell proliferation was examined by CCK8. (**B**, **C**) Cell migration and invasion were tested by Transwell assay. (**D**) Cell apoptosis was evaluated by FITC. **P*<0.05, ***P*<0.01 vs NC group.

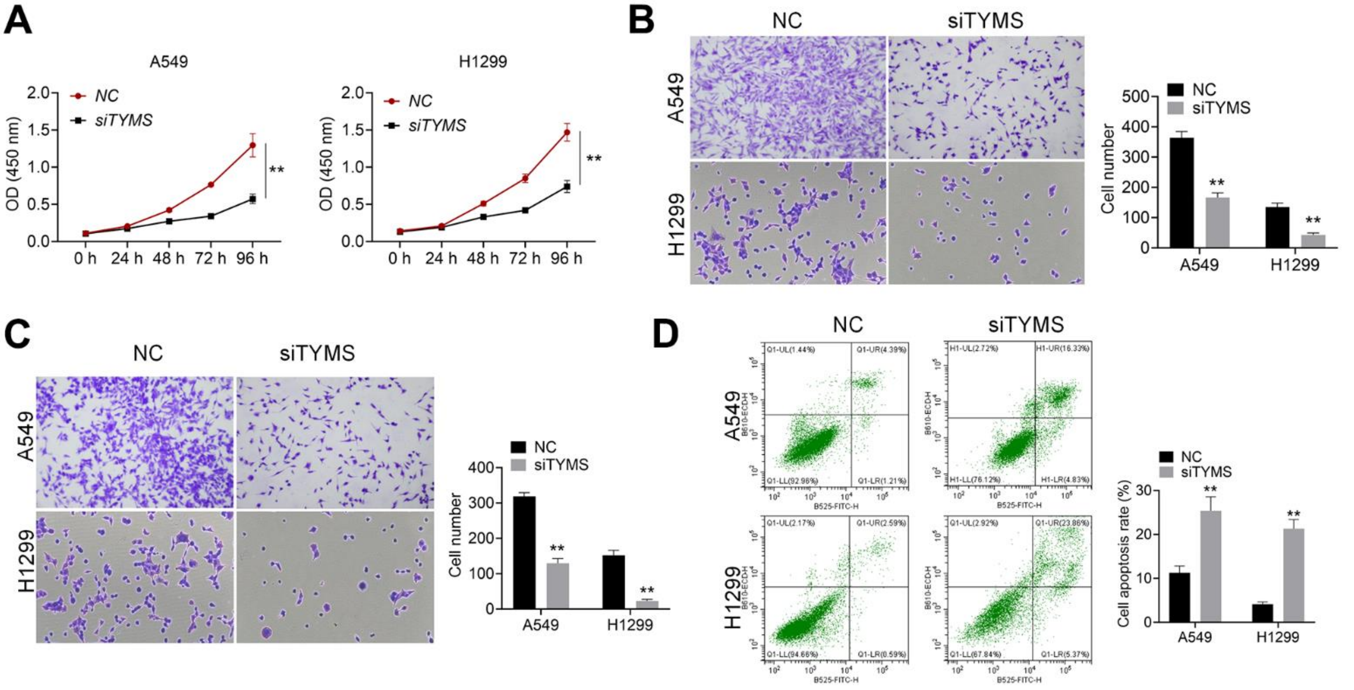

Supplementary Figure 4. Effect of TYMS knockdown on malignant oncology phenotype in NSCLC cells. (**A**) Cell proliferation was examined by CCK8. (**B**, **C**) Cell migration and invasion were tested by Transwell assay. (**D**) Cell apoptosis was evaluated by FITC. **P*<0.05, ***P*<0.01 vs NC group.
